# Prevalence and distribution of abdominal aortic plaque in a community-dwelling cohort free of clinical cardiovascular disease and major cardiovascular disease risk factors

**DOI:** 10.1186/1532-429X-16-S1-P102

**Published:** 2014-01-16

**Authors:** Michael L Chuang, Philimon Gona, Noriko Oyama-Manabe, Carol J Salton, Christopher J O'Donnell, Warren J Manning

**Affiliations:** 1 The NHLBI's Framingham Heart Study, Framingham, Massachusetts, USA; 2 Cardiovascular Division, Beth Israel Deaconess Medical Center, Boston, Massachusetts, USA

## Background

Abdominal aortic atherosclersosis is associated with incident cardiovascular disease (CVD); excess burden of atherosclerotic abdominal aortic plaque (AAP) may be useful for identification of persons at increased risk of occlusive vascular disease. We sought to determine the prevalence and quantitative burden of AAP detected by CMR in community-dwelling adults free of prevalent CVD and major CVD risk factors.

## Methods

1794 Framingham Heart Study (FHS) Offspring cohort members underwent ECG-gated, free breathing, fat suppressed, black-blood, T2 weighted turbo spin-echo CMR of the abdomen (24 slices with 1.03 × 0.64 × 5-mm3 voxel size, 5-mm interslice gap) at 1.5T (Gyroscan NT, Philips) over 2002-2006. AAP was defined as discrete protrusions of ≥1 mm into the aortic lumen and was manually planimetered (MASS v6.1, QT-Medis) for determination of plaque burden. Clinical characteristics were determined at the Offspring cycle visit immediately prior to CMR scanning (cycle 7, 1998-2001). We identified a referent group free of prevalent CVD, smoking, obesity (BMI<30 kg/m2), hypertension (BP < 140/90 mmHg, no antihypertensive medications), diabetes, and dyslipidemia (total cholesterol <240 mg/dL, no medications) and determined prevalence and burden of AAP by sex and by age group among referent participants.

## Results

Of the 1726 (96.2%) FHS Offspring with complete studies and adequate image quality, 525 (30.4%; 221 men, 304 women) met all referent-group criteria. Among these referent participants, overall prevalence of AAP was 34.8% in men and 42.4% in women (p = 0.095). Participants were stratified into four age groups as shown in the Table 1. Prevalence of AAP increased with greater age group in both sexes. Among those with AAP > 0, we determined sex-specific burden of AAP at the 25th, 50th, 75th and 90th percentiles (P25, P50, P75, P90 respectively) within each age group. Among referent men burden of AAP increased with greater age-group at each percentile cutpoint, but there was no clear pattern of increased AAP burden with greater age-group among referent women.

**Table 1:** 

			AAP Burden, cm3			
**Age Group**	**N**	**Prev AAP**	**P25**	**P50**	**P75**	**P90**

MEN						

<55 y	57	17 (30%)	0.14	0.30	0.40	2.20

55-64 y	88	32 (36%)	0.15	0.28	0.53	0.95

65-74 y	56	18 (32%)	0.18	0.41	0.61	2.74

≥75 y	20	10 (50%)	0.47	1.08	3.81	5.46

WOMEN						

<55 y	64	23 (36%)	0.18	0.52	1.30	1.75

55-64 y	143	58 (41%)	0.20	0.44	1.21	1.93

65-74 y	76	35 (46%)	0.22	0.41	1.20	2.21

≥75 y	21	13 (62%)	0.17	0.47	1.68	1.90

						

Prev AAP = prevalence of abdominal aortic plaque, P = percentile

## Conclusions

Abdominal aortic atherosclerosis is common even among adults free of clinical CVD and major CVD risk factors, with CMR evidence of AAP identified in 39% of such adults. Both prevalence and burden of AAP increased with greater age in men, but only prevalence increased with age in women. The prevalence of AAP appeared greater in women than men in each age group, but did not reach statistical significance in the overall sample. In younger age groups AAP burden was greater in women than men, but in the older age groups men had similar or greater burden of AAP. Whether this represents true sex-associated crossover in burden of total AAP with greater age, or is due to sex differences in distribution of AAP (i.e. generalized wall thickening versus discrete protrusions) remains to be determined.

## Funding

Supported by the National Heart, Lung and Blood Institute: (The Framingham Heart Study: N01-HC-25195 and N01-HC-38038) and by RO1 HL70279. Dr. O'Donnell is supported by the NHLBI Division of Intramural Research.

